# Sustained Release of Basic Fibroblast Growth Factor (bFGF) Encapsulated Polycaprolactone (PCL) Microspheres Promote Angiogenesis In Vivo

**DOI:** 10.3390/nano9071037

**Published:** 2019-07-20

**Authors:** Pala Arunkumar, Julie A. Dougherty, Jessica Weist, Naresh Kumar, Mark G. Angelos, Heather M. Powell, Mahmood Khan

**Affiliations:** 1 Department of Emergency Medicine, College of Medicine, Davis Heart and Lung Research Institute, The Ohio State University Wexner Medical Center, Columbus, OH 43210, USA; 2 Department of Materials Science and Engineering, Department of Biomedical Engineering, The Ohio State University, Columbus, OH 43210, USA; 3 Research Department, Shriners Hospitals for Children, Cincinnati, OH 43210, USA

**Keywords:** heart disease, angiogenesis, polycaprolactone, microspheres, basic fibroblast growth factor

## Abstract

Coronary heart disease (CHD) is the leading cause of death in the Unites States and globally. The administration of growth factors to preserve cardiac function after myocardial infarction (MI) is currently being explored. Basic fibroblast growth factor (bFGF), a potent angiogenic factor has poor clinical efficacy due to its short biological half-life and low plasma stability. The goal of this study was to develop bFGF-loaded polycaprolactone (PCL) microspheres for sustained release of bFGF and to evaluate its angiogenic potential. The bFGF-PCL microspheres (bFGF-PCL-MS) were fabricated using the emulsion solvent-evaporation method and found to have spherical morphology with a mean size of 4.21 ± 1.28 µm. *In vitro* bFGF release studies showed a controlled release for up to 30 days. Treatment of HUVECs with bFGF-PCL-MS *in vitro* enhanced their cell proliferation and migration properties when compared to the untreated control group. Treatment of HUVECs with release media from bFGF-PCL-MS also significantly increased expression of angiogenic genes (bFGF and VEGFA) as compared to untreated cells. The *in vivo* angiogenic potential of these bFGF-PCL-MS was further confirmed in rats using a Matrigel plug assay with subsequent immunohistochemical staining showing increased expression of angiogenic markers. Overall, bFGF-PCL-MS could serve as a potential angiogenic agent to promote cell survival and angiogenesis following an acute myocardial infarction.

## 1. Introduction

Cardiovascular diseases (CVD) are the leading cause of death globally [1]. In the Unites States (US) alone, Coronary heart disease (CHD) contributes to 43.8% of all deaths due to CVD. Furthermore, CHD is one of the ten most expensive medical conditions (total medical cost associated is ~$ 9 billion) treated in US hospitals in 2013 and it is projected that the cost will increase by ~100% between 2013 and 2030 [2]. The current treatment regimens mainly include administration of pharmacological drugs (anticoagulants, platelet inhibitors, cholesteryl ester transfer protein (CETP) inhibitors and β-blockers) [3,4], surgical interventions (implanting pacemaker [5], metallic/biodegradable drug-eluting stents [6], implantable cardioverter defibrillator (ICD) [7], coronary artery bypass graft surgery (CABG)) [8], and heart transplantation [9] in extreme cases. The two earlier strategies provide only symptomatic relief without addressing the causative mechanisms. In the case of heart transplantation, donor scarcity is one of the major challenges. To overcome these drawbacks, researchers are working towards developing novel bioengineering strategies to repair and regenerate the cardiac tissue after myocardial infarction (MI) [10,11]. One such strategy is to administer growth factors that are vital for the repair and regeneration of cardiac tissue [12]. Growth factors act as mitogens and play a potent role in angiogenesis, cell migration and cardioprotection [13,14,15,16,17,18].

Basic fibroblast growth factor (bFGF), also known as fibroblast growth factor-2 (FGF-2) and a member of the FGF family, regulates cell growth, differentiation, angiogenesis, tissue homeostasis, and tissue repair [19,20]. It is predominantly expressed in heart tissue at all developmental stages as well as in other cell types including endothelial cells, smooth muscle cells, and fibroblasts [21]. There are two isoforms of FGF-2: Low molecular weight FGF-2 (18 kDa), which is beneficial for an adaptive trophic response after MI [21]; while high molecular weight FGF-2 (20–34 kDa) is pro-hypertrophic and pro-apoptotic leading to maladaptive remodeling [22]. In this paper, bFGF represents only the low molecular weight isoform. The bFGF is a potent mitogen for a variety of cells (mesodermal and neuroectodermal origin, bone marrow-derived, and tissue-derived or resident human and rodent cells with stem-cell properties) and also a strong angiogenic agent [23,24,25,26,27,28]. Additionally, unlike the receptors for VEGF and other growth factors, FGF receptors are found to be expressed on both endothelial cells and smooth muscle cells, which contributes to the formation of a mature blood vessel network [29]. Moreover, bFGF is a more potent promoter of endothelial cell proliferation when compared to VEGF [30,31], hence bFGF is considered a superior candidate over VEGF. It has also been reported that bFGF plays a role in maintaining the self-renewal property of induced pluripotent stem cells (iPSCs) [32] and imparts cardioprotective properties after a cardiac injury [21,24,33,34]. Although there are several advantages of bFGF, the clinical response to bFGF treatment in larger randomized placebo-controlled clinical trials was limited due to its’ rapid diffusion, poor bio-stability and short half-life [35,36,37].

In order to overcome these drawbacks, sustained bFGF release systems using polymeric biomaterials like scaffolds, hydrogels and microspheres are being explored [38,39,40,41,42,43,44,45,46,47]. Among them, polymeric microspheres are preferred due to their ease of administration. There are reports on bFGF delivery using microspheres based on polymers like poly(lactic-co-glycolic acid) (PLGA) [43,48,49], chitosan [50,51], alginate [52], gelatin [47,53,54], and acetalated dextran [40]. Among the above list, all the polymers except for PLGA are of natural origin and have an inherent batch-to-batch variation as well as faster release kinetics. In contrast, PLGA microspheres are of synthetic origin, whose properties can be fine-tuned based on synthetic methods [55] and also exhibit controlled release. Unfortunately, the main concern with PLGA-based systems is the release of toxic acidic byproducts upon degradation *in vivo* [56].

Polycaprolactone (PCL) is yet another versatile synthetic polymer approved by the Food and Drug Administration (FDA) for drug delivery and suture applications [57]. PCL is widely used for the development of long-term controlled release systems [57,58]. As PCL is more hydrophobic than PLGA, it degrades slower than PLGA, so the rate of release of toxic acidic degradation products is slower and they are quickly cleared upon formation [56]. Moreover, the acidic product (caproic acid with pKa = 4.84) released upon PCL degradation is less acidic, when compared to the degradation products of PLGA: lactic acid (pKa = 3.08) and glycolic acid (pKa = 3.83) [56,59]. To the best of our knowledge, there are no reports on PCL microspheres as a controlled bFGF release system for inducing angiogenesis in the treatment of myocardial infarction or for cardiac tissue engineering. Therefore, the main goal of this work was to fabricate bFGF-loaded PCL microspheres, characterize their morphology and evaluate their angiogenic properties using both *in vitro* and *in vivo* models.

## 2. Materials and Methods

### 2.1. Materials

Recombinant Human FGF-basic (bFGF) was purchased from Peprotech, Rocky Hill, NJ, USA; Heparin Sodium Salt from Porcine Intestinal mucosa was obtained from EMD Millipore Corp, Danvers, MA, USA; Polycaprolactone (PCL) [Mw = 70,000], Polyvinyl alcohol (PVA) [87–90% hydrolyzed, average molecular weight ~30–70 k], Bovine Serum Albumin (BSA) [heat shock fraction, protease free, fatty acid free, ≥98%], fluorescein and rhodamine were purchased from Sigma-Aldrich, St. Louis, MO, USA; Geltrex^TM^ (LDEV-free reduced growth factor basement membrane matrix without phenol red) was obtained from ThermoFisher Scientific, Waltham, MA, USA; Chloroform, analytical grade, was purchased from Acros Organics, NJ, USA.

### 2.2. Fabrication and Characterization of bFGF Loaded PCL Microspheres

**Figure 1:**
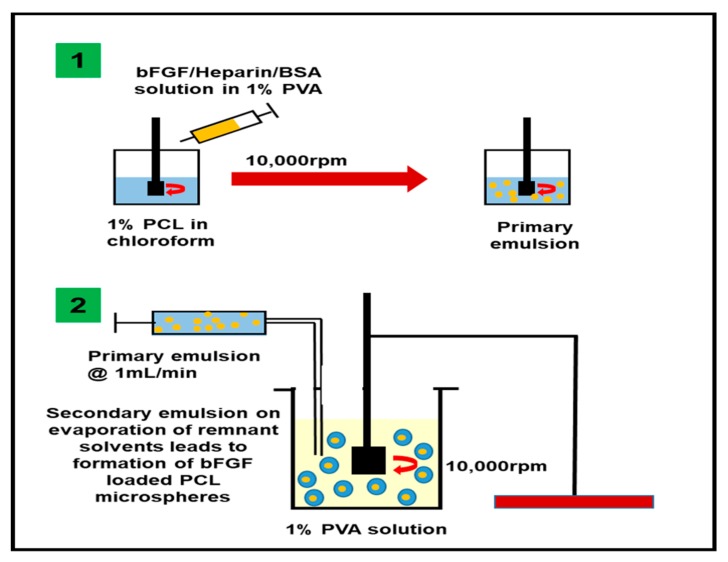
Schematic representation of w_1_/o/w_2_ emulsion solvent evaporation method to fabricate bFGF-PCL-MS.

The bFGF-loaded PCL microspheres (bFGF-PCL-MS) were fabricated using the w_1_/o/w_2_ emulsion solvent evaporation method, as per the protocol from previous literature [60], the schematic of which is shown in Figure 1. Briefly, 150 µg bFGF was dissolved in 1% PVA solution containing 0.5% Heparin and 0.1% BSA as stabilizers. 1 mL of this solution was emulsified under homogenization at 10,000 rpm into 10 mL of 1% PCL solution made using chloroform. The prepared primary emulsion was again added on to 1% PVA solution with a flow rate of 1 mL/min and homogenized at 10,000 rpm to prepare a secondary emulsion. The obtained secondary emulsion was then kept under magnetic stirring for 3 h to evaporate the remnant solvent and thus harden the formed microspheres. The solution containing microspheres was centrifuged thrice to wash the microspheres and remove the unbound bFGF. The obtained microsphere pellet was dispersed in 10 mL PBS and freeze-dried to obtain a lyophilized powder of bFGF-PCL-MS. To study the microspheres’ morphology, a few milligrams of microspheres were weighed, then dispersed and diluted in PBS. It was then added onto a small piece of aluminum foil sheet and dried at room temperature. Scanning electron microscopy (SEM) imaging (FEI Novs NanoSEM 400) was performed at an accelerating voltage of 5 kV after gold coating the particles using a sputter coater (Pelco Model 3 sputter-coater). The mean size was determined using randomly chosen 100 microspheres each from three different batches in triplicates and the analysis was performed using Image J.

To assess the distribution of the encapsulated contents within the microspheres, fluorescein and rhodamine were mixed with 1% PCL and the inner aqueous phase (containing 1% PVA, 0.5% Heparin and 0.1% BSA) solution, respectively, and the microspheres were fabricated as per the protocol above. The obtained microspheres were imaged using a confocal imaging system (Olympus FV 3000). The amount of bFGF encapsulated within the microspheres was determined using a bFGF ELISA assay kit (Peprotech, Rocky Hill, NJ, USA), using three independent batches according to the manufacturer’s protocol.

### 2.3. In Vitro bFGF Release Studies

The bFGF-PCL-MS powder (5 mg) was weighed and dispersed using 0.5 mL PBS in a 2 mL centrifuge tube, which was kept at 50 rpm in a shaking incubator maintained at 37 °C. At designated time intervals (day 1, 3, 5, 7, 14, 21, 28, and 31) the microspheres were centrifuged, the supernatant removed and collected for further analysis to quantify the amount of bFGF released using ELISA kit assay. Fresh PBS (0.5 mL) was added to the pellet and the above procedure was repeated at various time intervals for a month. The cumulative amount of bFGF released was expressed as mean ± SD, *n* = 3.

### 2.4. In Vitro Human Endothelial Cell Proliferation Studies

In order to study the effect of released bFGF from bFGF-PCL-MS on human umbilical vein endothelial cells’ (HUVECs) proliferation rate, bFGF-PCL-MS (5 mg/mL) were incubated for 48 h in two different types of media. One in which the media has 2% FBS and all the supplements needed for cell growth (as per the manufacturer’s protocol); and the other has 2% FBS without any supplements. This is done to isolate the effect of released bFGF on the cell proliferation rate with and without the supplements.

The *in vitro* cell proliferation assay was performed using XTT assay as per the manufacturer’s instructions. Briefly, cells were cultured in endothelial cell growth medium (VascuLife Basal Medium, LifeLine Cell Technology, Oceanside, CA, USA) supplemented with 2% (*v*/*v*) FBS and all of the supplements provided with the VascuLife VEGF Endothelial Medium Complete Kit (LifeLine Cell Technology, Oceanside, CA, USA). The cells were incubated at 37 °C with 5% CO_2_ and at a saturated humid atmosphere. Nearly confluent cells in 75 cm^2^ tissue culture flasks were trypsinized and re-suspended in fresh medium. The number of cells was counted using hemocytometry. The re-suspended cells were diluted accordingly and were plated at a concentration of 8,000 cells per well in a 96-well tissue culture plate and incubated in a CO_2_ incubator for 24 h. After 24 h, the wells were segregated into four different groups (each with *n* = 4) and treated with four different treatments: (1) bFGF release medium with all the supplements; (2) bFGF release medium with no supplements; (3) medium with all the supplements; (4) medium with no supplements. The cell proliferation rate was calculated using XTT assay at three different time points: immediately after the addition of all the treatments (Day 0), 24 h after the treatments (Day 1), and 48 h after the treatments (Day 2). The cell proliferation rate was then normalized with respect to the control group and represented. One-way analysis of variance (ANOVA) test was carried out in order to evaluate the statistical significance. Data is expressed as mean ± SD, * *p* < 0.001 was considered to have high statistical significance.

### 2.5. In Vitro Wound Healing/Scratch Assay to Assess Endothelial Cell Migration

HUVECs were seeded in a 12-well plate and grown to 100% confluence. The cell monolayer was wounded with a 200 µl pipet tip, washed twice with PBS and 900 µl of basal media was added per well. Light microscopy images were captured immediately at baseline (0 h). Treatment media included (a) Negative control-VascuLife Basal media (LifeLine Cell Technology, Oceanside, CA, USA) supplemented with 2% FBS; (b) bFGF-MS release media (media collected after incubation of bFGF-PCL-MS (5 mg/mL) for 48 h) supplemented with 2% FBS. Cell culture wells were imaged with EVOS™ FL Auto 2 Imaging System (ThermoFisher, Waltham, MA, USA) with six frames per well programmed into the scan protocol to capture the same fields over the course of the experiment. Images were captured at 10× magnification at 0, 3, 6, and 9 h. Wound area was analyzed with ImageJ by a blinded operator. Total area per well was calculated and percent wound closure was determined relative to time 0 with *n* = 4 wells per group. Data is expressed as mean ± SD, * *p* < 0.01 as compared to negative control at same time point was considered statistically significant.

### 2.6. Gene Expression Analysis of Growth Factors in HUVECs

HUVECs were incubated with negative control media or the release media from bFGF-PCL-MS for 48 h and total RNA was extracted using the Direct-zol RNA Miniprep kit, with on-column DNase digestion (Zymo Research, Irvine, CA, USA). 1 μg of total RNA was used as input for cDNA synthesis using the High Capacity Reverse Transcriptase Kit (Applied Biosystems, CA, USA) as per the manufacturer’s instructions. TaqMan assays (ThermoFisher, Waltham, MA, USA) were used to analyze expression of growth factors bFGF (Hs00266645_m1) and VEGFA (Hs00173626_m1) along with 2× TaqMan Universal PCR Mix (Applied Biosystems, CA, USA). Reaction conditions were according to the manufacturer’s instructions and samples were analyzed in triplicate wells. SYBR green detection was used with primers to analyze EC markers CD31 (F 5′-AACAGTGTTGACATGAAGAGCC-3′; R 5′-TGTAAAACAGCACGTCATCCTT-3′) and vWF (F 5′-CCGATGCAGCCTTTTCGGA-3′; R 5′-TCCCCAAGATACACGGAGAGG-3′). RPL13a acted as the housekeeping gene for all comparisons (F 5′-GCCTACAAGAAAGTTTGCCTAT-3′; R 5′-CTTCTTCCGGTAGTGGATCTT-3′). SYBR green reaction conditions were: 95 °C 10 m→ (95 °C 10 s→60 °C 10 s→72 °C 20 s*) × 40 cycles (* denotes fluorescent detection). Reactions were run on a QuantStudio 3 (ThermoFisher, Waltham, MA, USA) and Ct values calculated by the QuantStudio Design & Analyze software (v1.4.1, ThermoFisher, Waltham, MA, USA). Data were normalized to RPL13a and fold expression was calculated relative to negative control using the 2^-ΔΔCt^ method [61], with efficiency correction where necessary [62]. Data represents mean ± SD (*n* = 3).

### 2.7. In Vivo Angiogenesis Assay

The *in vivo* feasibility study to assess the angiogenic potential of the microspheres was evaluated in an eight week old Sprague Dawley rat (150–200 g) using *in vivo* Matrigel plug angiogenesis assay as per the guidelines of Institutional Animal Care and Use Committee (IACUC), The Ohio State University (NIH Publication No. 86-23). It was performed by subcutaneous injection of Geltrex (growth factor reduced basement membrance matrix, Gibco, Gaithersburg, MD, USA) mixed with and without microspheres. Briefly, bFGF-PCL-MS (50 mg) were mixed with 500 µL Geltrex and injected subcutaneously in the flank region. Geltrex without microspheres were injected as control. The left side of the animal’s abdomen was injected with control while the right side was injected with the sample. Geltrex plugs were allowed to incubate inside the body for a week. After a week, the animal was euthanized and the plugs were surgically removed, washed well and imaged. Subsequently, the plugs were fixed with 4% paraformaldehyde, cryo-frozen with OCT embedding compound, and sectioned (5 µm each) using a cryostat. Immunohistochemistry staining (for two angiogenic biomarkers: lectin and α-SMA) was performed on the sections as per the protocol established in the earlier studies [63]. Briefly, slides were rinsed with distilled water, permeabilized with 0.1% Triton-X in PBS for 10 m, and blocked with 10% normal goat serum for 1 h. Sections then underwent double immunofluorescent staining with a FITC-lectin conjugate (1:200, Griffonia Simplicifolia Lectin I from Trevigen, Gaithersburg, MD, USA) and α-SMA (1:500, Millipore-Sigma, St. Louis, MO, USA) and incubated for 1 h with corresponding secondary antibody: Alexa Fluor 594 conjugate (1:1000, Cell Signaling Technologies, Danvers, MA, USA). Slides were mounted with ProLong Glass Antifade Mountant (ThermoFisher, Waltham, MA, USA) and then imaged using a confocal microscope. (Olympus FV 1000 spectral, Olympus Corporation, PA, USA). Image analysis was performed using Olympus FLUOVIEW Ver. 4.2 a Viewer. The number of new capillaries per high power field (HPF) was manually quantified using four images each for the slides prepared from both the control and treated plugs. Unpaired t-test with Welch’s correction was used to compare the capillary density between the control and the treatment group. A value of * *p* < 0.001 was considered to be statistically significant.

## 3. Results

### 3.1. Fabrication of bFGF-PCL-MS

**Figure 2:**
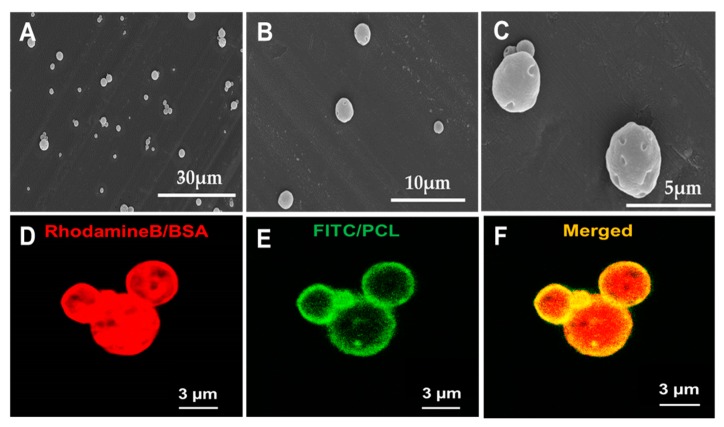
SEM images of bFGF-PCL-MS with three different magnifications (**A**) 2400× (**B**) 6500× and (**C**) 10,000×. Confocal microscopy images of fluorescent PCL-MS. (**D**) RhodamineB/BSA distribution in the microspheres. (**E**) FITC/PCL distribution in the microspheres. (**F**) Merged image.

The bFGF-PCL-MS were successfully fabricated using w_1_/o/w_2_ double emulsion solvent evaporation method. Microspheres were found to be spherical in shape with minor deformations on their surface (Figure 2A–C). The microspheres were found to have a mean diameter of 4.21 ± 1.28 µm. Confocal microscopy of the fluorescently labeled microspheres confirmed that most of the encapsulated contents (in red) were found to be distributed within the green polymeric shell, with some distributed on the surface as well (Figure 2D–F). This indicated that the majority of the encapsulated bFGF is entrapped within the microspheres. The amount of bFGF loaded was found to be 3.9 ± 0.3 ng per mg of microspheres.

### 3.2. In Vitro Release Studies

**Figure 3:**
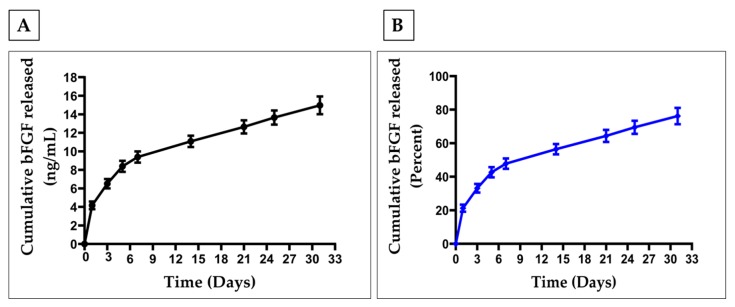
In vitro bFGF release profile of bFGF-PCL-MS. (**A**) Cumulative bFGF released in ng/mL (**B**) Cumulative bFGF released in percentage.

The *in vitro* release profile showed an initial burst release of bFGF followed by a sustained release for almost a month’s time (Figure 3A,B). It was observed that about 20% (−4 µg/mL) and 33% (6.5 µg/mL) of the total bFGF were released on day 1 and 3, respectively. At the end of 21 days, almost 60% of total bFGF was released, followed by −77% of bFGF released at the end of a four weeks’ time.

### 3.3. In Vitro Cell Proliferation Studies

**Figure 4:**
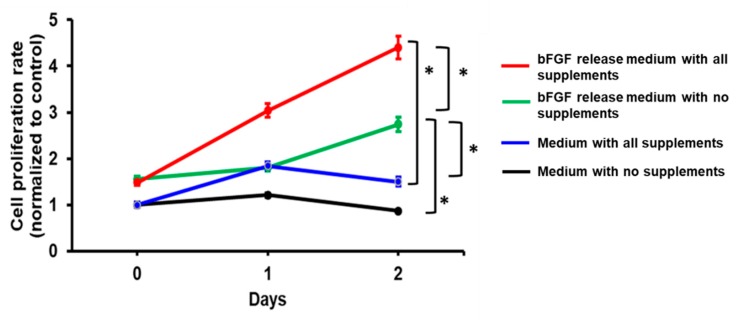
*In vitro* cell proliferation studies using the bFGF release medium collected after 48 h-incubation of bFGF-PCL-MS. * *p* < 0.001 is considered significant.

The bFGF released from the bFGF-PCL-MS was active and stimulated the growth of HUVEC in an *in vitro* cell proliferation assay (Figure 4). HUVECs incubated with the bFGF-release media for 24 h and 48 h had significantly higher proliferation rate when compared to control media (no supplements). It was interesting to observe that cells grown on the bFGF release media with and without supplements had a higher proliferation rate (2 and 3-fold higher, respectively) when compared to their respective controls. Moreover, among the bFGF release media treated groups, cells treated with media containing supplements had significantly higher proliferation rate when compared to the ones without them. Furthermore, cells treated with bFGF release media without supplements showed significantly increased proliferation as compared to cells with fully supplemented media. This data indicated that the released bFGF had a significant effect on the cell proliferation rate and acted as a potent mitogenic agent for the endothelial cells.

### 3.4. In Vitro Wound Healing/Scratch Assay for Assessing Endothelial Cell Migration

**Figure 5:**
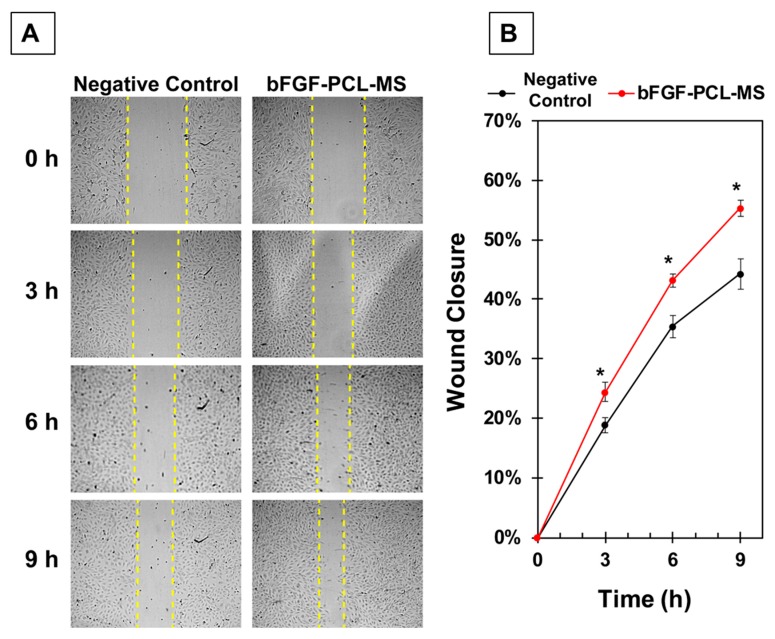
(**A**) Representative images comparing the cell migration between the treated and untreated groups at various time points. (**B**) Quantification of endothelial cell migration in terms of wound closure (in percentage) for both the treated and untreated groups at various time points. * *p* < 0.01 as compared to negative control.

Next, we examined whether the bFGF-PCL-MS release media could stimulate endothelial cell migration, a key step in angiogenesis, using a wound healing/scratch assay. HUVECs were grown to 100% confluence and wounded with a pipet tip. Wells were imaged at 0, 3, 6, and 9 h after the scratch was made and wound area was measured and compared to the time 0 h area. Figure 5A shows representative images comparing the cell migration between the treated and untreated groups at various time points. Results demonstrated that bFGF-PCL-MS release media significantly increased endothelial cell migration at all three time points as compared to negative control treatment as shown in Figure 5B. Overall, the results demonstrate that, bFGF-PCL-MS particles are capable of imparting angiogenic effects on endothelial cells.

### 3.5. bFGF-PCL-MS Release Media Stimulates Cellular Expression of Angiogenic Genes

**Figure 6:**
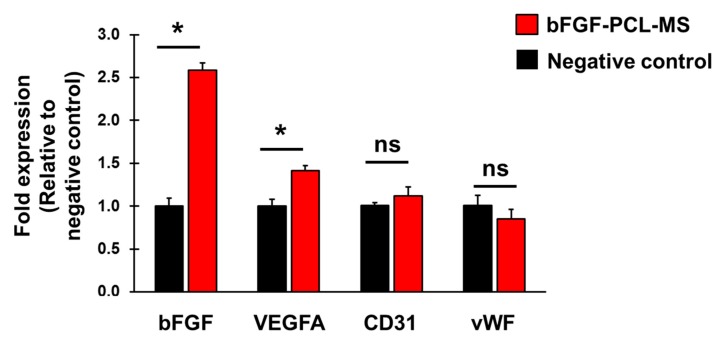
bFGF-MS release media enhances endothelial cell growth/angiogenic factor gene expression. Gene expression of HUVEC cells incubated with negative control or bFGF-PCL-MS release media for 48 h. Angiogenic factors (bFGF, VEGFA) and endothelial cell markers (CD31, vWF) were analyzed by qPCR with expression calculated relative to negative control. Data represents mean ± SD (*n* = 3), wound closure as compared to 0 h.* *p* < 0.01 vs negative control.

HUVECs were treated with negative control media or bFGF-PCL-MS release media for 48 h and gene expression was analyzed using qPCR. Cellular expression of bFGF increased significantly (2.6-fold) in treated cells as compared to negative control (Figure 6). Additionally, expression of VEGFA also increased significantly (1.4-fold) in treated cells as compared to negative control (Figure 6). We also analyzed expression of endothelial cell markers CD31 and vWF, however, there was no significant change in their expression (Figure 6). These results demonstrate that treating HUVECs with bFGF-PCL-MS release media increases cellular expression of angiogenic growth factors and does not alter their endothelial cell identity.

### 3.6. In Vivo Angiogenesis Assay

**Figure 7:**
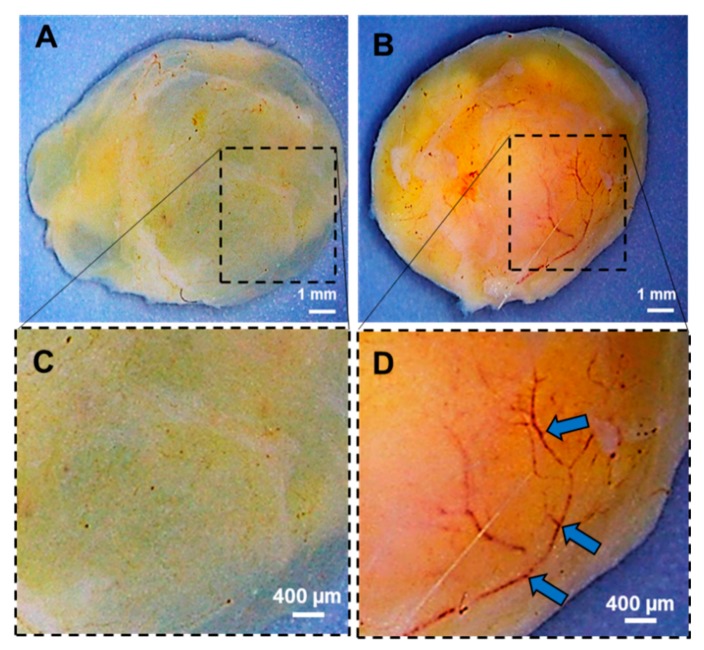
*In vivo* matrigel plug assay to evaluate the angiogenic potential of bFGF-PCL-MS. (**A**) Control (Only Geltrex) (**B**) bFGF-PCL-MS treatment (Geltrex + bFGF-PCL-MS). (**C**,**D**) are zoomed in images of A and B respectively. The arrows (Blue) indicate the new prominent blood vessels formed in the Geltrex plug of bFGF-PCL-MS treated rat.

**Figure 8:**
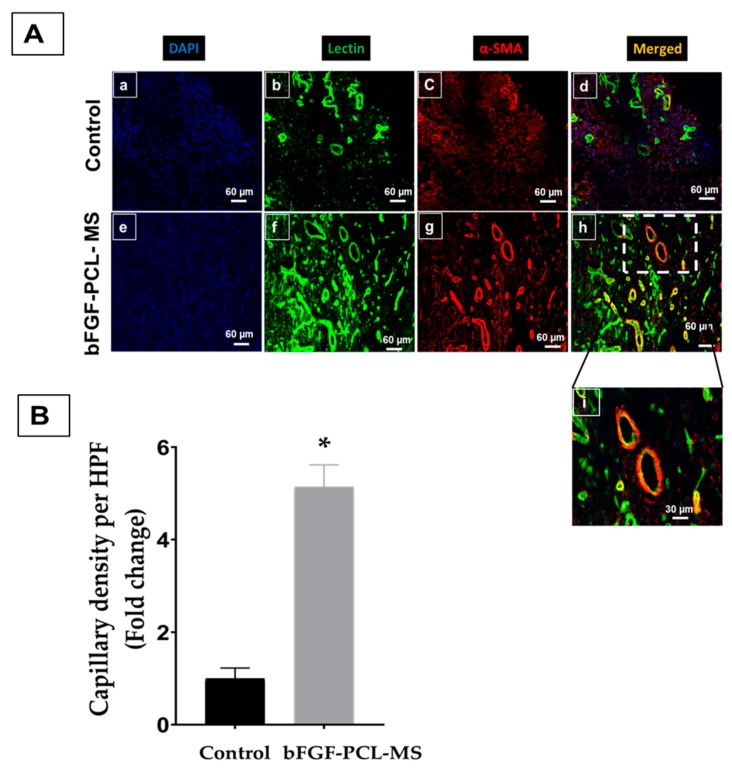
(**A**) Neovascularization as imaged and quantified by immunostaining for lectin and α-SMA. Confocal imaging of implanted Matrigel sections at one-week demonstrated enhanced angiogenesis in the bFGF-PCL-MS treated plug when compared to the control. (**a**–**d**) represents the control (no treatment) rat; (**e**–**h**) represents the bFGF treatment. Scale bar: 60 µm. (**i**) represents the zoomed in image of (**h**) showing two fully matured blood vessels. (**B**) Quantification of the number of new blood vessels formed per high power field in the bFGF-PCL-MS treated plug when compared to the control. Images (*n* = 4) were quantified and data normalized to the control rat. All values were expressed as mean ± SD, * *p* < 0.001 as compared to control group.

Angiogenesis was assessed via an *in vivo* Matrigel plug assay by injecting Geltrex subcutaneously in the rat’s flank region. Geltrex with bFGF-PCL-MS showed an increased vessel formation in the plug at one-week when compared to the control (Figure 7), and a network of vessels are clearly visible in the bFGF-PCL-MS treated plug (Figure 7D). Furthermore, immunohistological staining of angiogenesis markers (lectin and α-SMA) confirmed the increased neovascularization in the bFGF-PCL-MS containing plug when compared to the control (Figure 8A,B). These results strongly demonstrated the *in vivo* angiogenic potential of bFGF-PCL-MS.

## 4. Discussion

Ischemic heart disease causes decreased supply of nutrients and oxygen to the heart muscle due to the blockage of the artery and leads to irreversible necrosis of cardiac tissue. Furthermore, enhancement of angiogenesis and improvement in collateral circulation are currently being explored as key strategies to repair and regenerate injured myocardium [64,65]. Although growth factors are found to have significant angiogenic properties, their efficacy is low due to their short half-life and poor bio-stability *in vivo* [35,36,37]. The angiogenesis process occurs in three different ways: vasculogenesis, angiogenesis, and arteriogenesis [65,66,67,68]. Vasculogenesis involves the process of differentiation and replication of endothelial progenitor cells to form an undeveloped vasculature. In angiogenesis, a new vascular network is formed from the already existing capillaries by the proliferation and migration of endothelial cells. Thus, typically only endothelial cells participate in the case of both vasculogenesis and angiogenesis, resulting in the formation of only small and premature arteries [65,66]. On the contrary, arteriogenesis involves recruitment of smooth muscle cells as well as vessel enlargement through vascular remodeling which results in maturation and stabilization of already existing arterioles [68,69]. Thus, a growth factor that induces arteriogenesis is considered optimal for the induction of large-conductance collateral vessels [47]. Recent studies have confirmed a significant role of bFGF in enhancing angiogenesis as well as arteriogenesis in ischemic tissues [70,71]. Therefore, bFGF is considered as an ideal growth factor candidate for therapeutic revascularization, hence we have selected it for *in vitro* and *in vivo* experiments in our current study.

Polymeric microspheres are explored as a controlled release system for bFGF and thus could improve its stability and half-life [40,47,48,49,50,53]. We fabricated bFGF-PCL-MS to meet the same objective of improving the stability and half-life of bFGF. The bFGF-PCL-MS in our study were found to have spherical morphology with a mean diameter of 4.21 ± 1.28 µm, which is considered a safe size range for injection without blocking any arteries. The microspheres were found to release bFGF in a controlled manner for a month after a brief initial burst release phase, as shown in Figure 3. The amount of bFGF loaded was found to be 3.9 ± 0.3 ng per mg of the microspheres. The reason for such a low bFGF loading amount might be due to the high hydrophobic nature of PCL [57] as bFGF, due to its hydrophilic nature, tends to move away from the polymer. This low loading amount is not uncommon in the literature. There are reports of low bFGF loading amounts when PLGA was used as a formulation polymer. For instance, bFGF-loaded porous PLGA microspheres were fabricated with a mean diameter of 50.80 ± 8.40 µm. The bFGF loading amount of porous PLGA microspheres with and without heparin immobilization was found to be 1124 ± 103 pg/mg and 270 ± 56 pg/mg, respectively. These values are lower than the loading amount obtained in this study. The heparin immobilized PLGA microspheres had a lesser initial burst release when compared to the microspheres without heparin [72]. Zou et al. developed bFGF-loaded PLGA microspheres having a mean diameter of 1.14 ± 0.30 µm with an initial burst release of 25.2% in day 1 followed by a much more sustained release (82%) until day 28 [43]. Similarly, bFGF-loaded chitosan microspheres were fabricated with a mean diameter of 35.34 µm. The amount of bFGF loaded was found to be 7.57 mg/g microspheres [51], which is higher than that obtained in this study. This could be because of two reasons. First, since both bFGF and chitosan are hydrophilic and in the aqueous phase, there is more chance for them to interact effectively. Secondly, the use of crosslinker sodium tripolyphosphate to form the chitosan network might have helped in trapping bFGF within the chitosan network. In another study, bFGF-loaded chitosan-gelatin microspheres were used with a mean diameter of 4.60 ± 2.28 µm loaded with 20 ng bFGF/mg of microspheres [50]. The higher loading amount, when compared to the present study, might be due to the similar reason explained above for the chitosan microspheres. Ali et al. fabricated alginate: collagen (in the ratio of 1:1) microspheres using a coaxial air jet method and the microspheres were found to have a mean diameter between 30 and 780 µm by varying a few process parameters. The microspheres (with a mean size of 30 µm) loaded with bFGF showed an initial burst release followed by a sustained release for seven days [52].

The *in vitro* cell proliferation and migration studies were performed in HUVECs, as it is a well-established model cell line system for angiogenesis-related studies [73,74]. The released bFGF was found to be effective in enhancing the cell proliferation and migration rates even in the absence of normal media supplements. Similarly, the *in vivo* matrigel plug assay confirmed the arteriogenic potential of the bFGF-loaded PCL microspheres. The immunohistochemical analysis on the sections obtained from the Matrigel plugs (Figure 8) showed an increased expression of both angiogenic markers, α-SMA and lectin. We had performed lectin and α-SMA staining to assess both the endothelial as well as smooth muscle markers in the newly formed blood vessels. The α-SMA stains the smooth muscle actin of the smooth muscle cells in the vessel wall, while lectin stains the inner surface of endothelial cells, which are present in the tunica intima or the innermost surface of blood vessels. We observed two different structures in the immunofluorescence analyses: the ones with red/green superposition (Figure 8i) indicated mature blood vessels with both endothelial as well as smooth muscle cells, while the others with only green fluorescence indicated newly formed blood vessels. The bFGF-PCL-MS treated plug had increased expression of both of these structures, which indicated that the released bFGF influenced maturation and formation of new vessels. Thus, our findings go hand-in-hand with the existing literature that bFGF can enhance the migration and proliferation of both the smooth muscle cells and the endothelial cells thus leading to the development of matured vessels [23,24,25,29]. Additionally, expression of angiogenic growth factors bFGF and VEGFA in HUVECs treated with bFGF-PCL-MS release media increased significantly as compared to untreated cells, pointing towards angiogenic potential of bFGF releasing microspheres. Overall, the bFGF-PCL-MS showed positive results both in the *in vitro* cell studies and *in vivo* angiogenesis studies. Future studies will be performed to assess angiogenesis by injecting these microspheres into the ischemic heart in a model of acute myocardial infarction. These microspheres can also be combined with stem cells to evaluate their effect on enhancing stem cell survival in the infarct heart. In addition to these, as angiogenesis is a very complex process involving the role of multiple growth factors, it will be interesting to study the possibility of delivering multiple growth factors either together or in a sequential manner and evaluate their positive effect on enhancing collateral vessel formation.

## 5. Conclusions

The bFGF-loaded PCL microspheres were successfully fabricated using w_1_/o/w_2_ emulsion solvent evaporation method. The *in vitro* bFGF release studies showed controlled release of bFGF for up to four weeks. The *in vitro* cell proliferation and migration assays on human endothelial cells further demonstrated that the released bFGF had significant angiogenic properties, with increased cellular expression of angiogenic factors as well. Similarly, the *in vivo* matrigel plug assay confirmed the enhanced angiogenic potential of bFGF-PCL-MS with an increase in neovascularization. Overall, we conclude that bFGF-PCL-MS could serve as a potential angiogenic agent to promote cell survival and angiogenesis following an acute myocardial infarction.
